# Building of Longitudinal Ultrasonic Assisted Turning System and Its Cutting Simulation Study on Bulk Metallic Glass

**DOI:** 10.3390/ma13143131

**Published:** 2020-07-14

**Authors:** Shuo Shan, Pingfa Feng, Huiting Zha, Feng Feng

**Affiliations:** 1 Division of Advanced Manufacturing, Graduate School at Shenzhen, Tsinghua University, Shenzhen 518055, China; ss17@mails.tsinghua.edu.cn (S.S.); feng.feng@sz.tsinghua.edu.cn (F.F.); 2 Lab of Intelligent Manufacturing and Precision Machining, Tsinghua Shenzhen International Graduate School, Tsinghua University, Shenzhen 518055, China; fengpf@mail.tsinghua.edu.cn; 3 State Key Laboratory of Tribology, Department of Mechanical Engineering, Tsinghua University, Beijing 100084, China

**Keywords:** bulk metallic glass, ultrasonic assisted turning, finite element analysis, cutting force

## Abstract

Bulk metallic glass (BMG) is a new kind of material which is made by rapid condensation of alloy. With excellent properties like high strength, high hardness, corrosion resistance, BMG is increasingly applied in mold manufacturing, weapon equipment and other fields. However, BMG is also one of hard-to-machine materials, which is arduous to be processed precisely and efficiently by the means of conventional cutting. Compared with conventional cutting, ultrasonic machining has a multitude of technological advantages such as reducing the cutting force, extending the tool life, etc. In ultrasonic machining, the ultrasonic electric signal is transformed into high frequency mechanical vibration on the tool, which changes the relationship between the tool and the workpiece in the process of machining. In this study, the longitudinal ultrasonic assisted turning (LUAT) system is established for processing BMG. Its resonant frequency and vibration characteristics are first simulated by modal analysis and harmonic response analysis, and then tested by displacement testing experiments, so that the suitable frequency and the amplitude for BMG turning can be selected and verified. On this basis, the two-dimensional turning finite element model is established to study the effect of ultrasonic vibration on cutting force under different cutting speeds. The research manifest that during the BMG turning, the assistance of longitudinal ultrasonic vibration can significantly reduce the average cutting force as well as the von Mises stress when the turning speed is below the critical turning speed. In addition, the tip of the tool contacts the workpiece discontinuously during cutting process which makes the instantaneous turning force in LUAT more periodic than that in conventional turning (CT).

## 1. Introduction

Bulk metallic glass (BMG), also called amorphous alloy or liquid metal because of its topologically disordered constituent atoms, is formed by the rapid condensation of alloy. In BMG, the absence of crystal defects such as grain boundaries and dislocations brings to a series of unique properties [1]. BMG has high strength, high hardness, low thermal expansion coefficient, low density and favorable corrosion resistance, which makes it a potential new engineering material [2,3,4,5]. However, these excellent properties of BMG on the other hand would lead to poor machinability. For example, during the cutting process of BMG, the cutting force is considerately large, which can damage the cutting tools, jeopardize the surface quality and hinders their wide scale application [6,7,8]. Karaguzel et al. [9] carried out the orthogonal cutting experiment of BMG, and the cutting force empirical formula of BMG is fitted by the experimental results and process parameters. Fujita et al. [10] examined cutting characteristics of BMG by tuning with different parameters, presumed a slipping-off mechanism at planes of short intervals of BMG. When cutting Zr-based BMG in low speed, there will be adiabatic shear bands and cavities in serrated chips, while in cutting with high cutting speed or small rake angle tools, there will be sparks and chip oxidation in the material [11,12,13,14]. Under ambient temperature, when BMG breaks, the local temperature of the alloy exceeds the glass transition temperature or even the melting point [15]. It is of great significance to raise new processing methods or improve the processing technology of BMG for promoting its engineering application.

Ultrasonic assisted machining has been termed as one of hybrid process that uses ultrasonic vibration during the machining action. Ultrasonic electric signal is converted into high-frequency mechanical vibration by transducer. The mechanical vibration is amplified by horn and transmitted to the end of the tool. In the turning process, the relative velocity between the tool tip and the workpiece changes due to the effect of ultrasonic vibration. Lauwers et al. [16] investigated the ultrasonic assisted grinding of ceramics, showed that the vibration resulted in more craters and lowered process force, making it feasible to increase the productivity. Pujana et al. [17] carried out ultrasonic assisted drilling experiments on titanium alloy, the results manifested that the drilling force decreases about 20%, and the it decreases with the increase of amplitude. Sui et al. [18] examined high-speed ultrasonic cutting, and the results showed that the material removal rate of high-speed ultrasonic cutting can be improved by up to 90% compared with conventional cutting. Maurotto et al. [19] applied ultrasonic longitudinal vibration to titanium alloy cutting. The average cutting force could be reduced by 70% compared with that of conventional cutting. Nath et al. [20] conducted cutting experiments on tungsten carbide by using elliptical ultrasonic vibration machining, studied the influence of tool nose radius on the surface quality, and obtained a better machining surface at 0.6-mm tool nose radius. Due to the application of longitudinal ultrasonic vibration, when the instantaneous speed of high-frequency vibration of the tool tip is greater than the cutting speed, there is contact-separation phenomenon between the tool tip and the workpiece. This kind of discontinuous contact makes the extrusion of the tool to the workpiece become high-frequency hammering, which changes the cutting force, cutting temperature, chip morphology and surface morphology of the machining surface.

A host of researches showed that ultrasonic vibration can improve the processing effect of BMG. Luo et al. [21] applied ultrasonic assisted micro-punch to BMG. The certain areas of BMG are subjected to the high frequency vibration transmitted by the punch, which leads to viscous flow. BMG gradually become soft and a series of shapes and products can be successfully fabricated in relative low pressing force. Ma et al. [22] proposed ultrasonic assisted forming for BMG processing, which not only forms BMG rapidly so that the crystallization and oxidation can effectively be avoided, but also works in a large scale range. In ultrasonic assisted punching and ultrasonic assisted forming on BMG, the cutting force and cutting temperature are effectively restrained, and the surface quality and productivity are improved. Whereas at present, there are few studies in ultrasonic assisted turning on BMG, which is subject to further investigation.

In this study, a longitudinal ultrasonic assisted turning (LUAT) system is established for processing BMG at first, and its frequency and amplitude are obtained through modal analysis and harmonic response analysis. A displacement testing experiment is performed to verify the designed system. Then the critical turning speed of the device is calculated. On this basis, the turning model of BMG is established, and the turning simulation of BMG under different turning speeds is carried out by using the LUAT device mentioned above. Finally, the influence of longitudinal ultrasonic vibration on the turning force, von Mises stress and chip forming under different turning speeds during turning BMG is discussed.

## 2. Building of Longitudinal Ultrasonic Assisted Turning System

### 2.1. Design of the Piezoelectric Transducer and the Ladder Horn

When a force is applied to the piezoelectric crystal in a proper direction, its internal polarization state will change. An internal electric field will then be generated, and a bound charge proportional to the external force will appear on two surfaces of the piezoelectric crystal. On the contrary, when an external electric field is applied to the piezoelectric crystal, the electric energy can be converted into mechanical energy through the piezoelectric crystal due to the inverse piezoelectric effect. The piezoelectric transducer is made of this inverse piezoelectric effect. Piezoelectric transducer has no eddy loss, hysteresis loss and resistance loss, which brings it fairly simple structure and high stability. Moreover, piezoelectric transducer usually has high sensitivity as well as outstanding electromechanical coupling characteristics. All these superior properties make piezoelectric transducer the most popular transducer in ultrasonic machining industry. In this investigation, a piezoelectric transducer with Lead Zirconate Titanate (PZT) is designed to convert electrical signals into ultrasonic vibration. Parameters of PZT are presented Table 1.

The longitudinal sound velocity in PZT slices $c_{PZT}$ can be calculated in Equation (1).

(1) $$c_{PZT}=\sqrt{\frac{E}{\rho }\frac{1}{1-\sigma^{2}}}$$

where, E is young’s modulus, ρ is density and σ is Poisson’s ratio of PZT-8.

The wavelength in PZT slices $\lambda_{PZT}$ could then calculated by $\lambda_{PZT}=c_{PZT}/f$. According to the design requirements of the longitudinal vibration transducer, the equivalent diameter of the PZT slices $D_{PZT}$ should less than $\lambda_{PZT}/4$, considering the inner holes, set the $D_{PZT}$ as 38 mm.

The output power of the PZT transducer is determined by the volume of PZT ceramic slice and its power capacity. In order to amplify the output power to satisfy the turning process, several pieces of PZT slices are piled. The number $m_{PZT}$ could be acquired by Equation (2).

(2) $$m_{PZT}=\frac{P}{P_{d}f\pi \left(D^{2}-d^{2}\right)\frac{t}{4}}$$

where $P_{d}$ is power capacity in W/cm^3^, *f* is vibration frequency and *D*, *d* and *t* represent outside diameter, inside diameter and thickness of ceramic slice, respectively.

**Figure 1:**
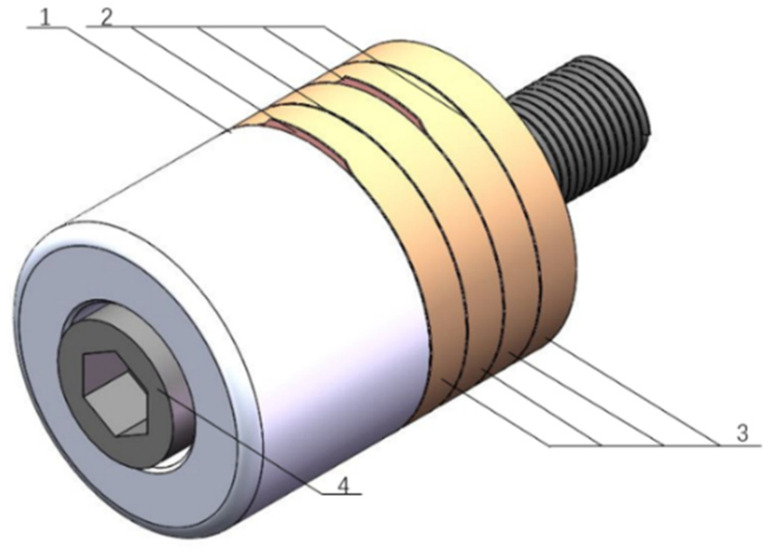
Structure diagram of the PZT transducer. 1. Back block; 2. electrodes; 3. PZT ceramics; 4. connect bolt.

The power capacity of PZT ceramic slice $P_{d}$ is 2–3 W/cm^3^, the required output power *P* is set as 800 W, considering the slices need to be piled in pairs, set the number of slices as 4. Four pieces of PZT slices are piled with copper electrodes separating between them, the polarization of two adjacent slices are opposite so that the pile could output an overlapped amplitude. The whole transducer used in this investigation is shown in Figure 1.

The output vibration amplitude the piezoelectric transducer is much less than required. To amplify the amplitude and to install the turning tool, a two-stage ladder horn is designed. The back end of the horn is contact with the PZT slices, so the diameter of the back end $D_{1}$ is 38 mm. The front end is designed much smaller than the back end and the energy of ultrasound is concentrated on this comparative smaller area. Due to the energy gathering effect, the front end of the horn outputs the amplified amplitude. The diameter of the front end $D_{2}$ determines the amplification *M*. The less $D_{2}$ is, the higher *M* will be. However, the strength may decrease when $D_{2}$ is reduced because the horn can be seen as cantilever structure during turning process and the components of cutting force can lead to enormous stress concentration on abrupt change in section. To ensure the enough strength and satisfy the amplification, set $D_{2}$ as 10 mm. Thus, the area index is $N=D_{1}/D_{2}=3.8$.

Longitudinal sound wave will bounce back and forth between the back and front end, the standing-wave effect happens when the incident wave and the reflected wave overlap. At specific points, the amplitude can be decreased to zero. These points can be called as displacement node, which can be used to fix the horn to the machine tool. Likewise, the amplitude reaches its peak at particular points, where the turning tool should be installed. The displacement node and the peak point can be acquired though wave equation which is shown in Equation (3).

(3) $$\frac{\partial^{2}\xi }{\partial x^{2}}+\frac{1}{S}\times \frac{\partial S}{\partial x}\times \frac{\partial \xi }{\partial x}+k^{2}\xi =0$$

where, ξ=ξ(x) is particle displacement function, S=S(x) is cross-section area function, $k=\omega /\sqrt{E/\rho }$ is circle wavenumber, ω is angular frequency, E is elastic modulus, ρ is the density of the horn.

In this investigation, the material of horn is 1045 steel, the parameters are listed in Table 2. Therefore, resonance length δ can be acquired as 258 mm, the length of the horn l is half of δ so that the front end located on the peak of the standing wave, outputting the largest amplitude.

**Figure 2:**
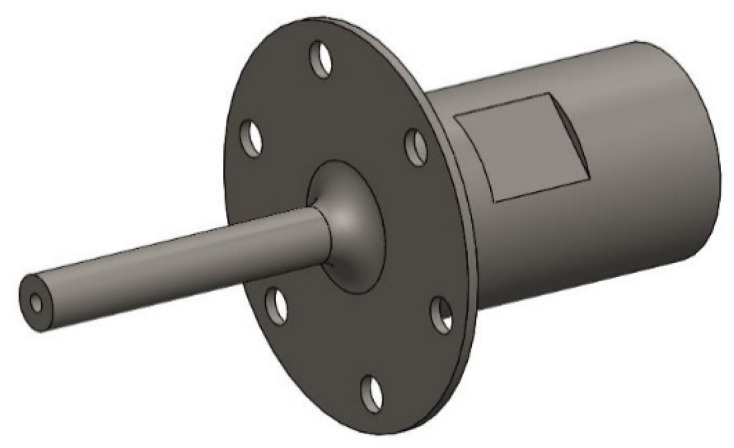
Two-stage ladder horn.

The displacement node located at the middle of the horn, where a flange plate is designed to install the horn to the machine tool. The ladder horn is shown in Figure 2.

### 2.2. Modal and Harmonic Analysis of the LUAT System

**Figure 3:**
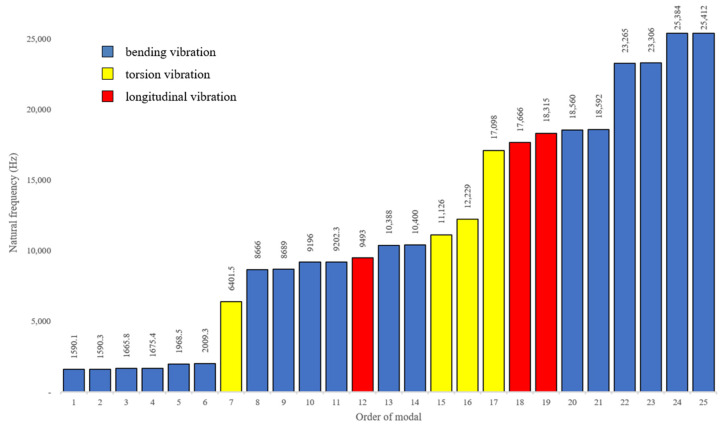
Natural frequency and vibration type of 25 orders of modals.

**Figure 4:**
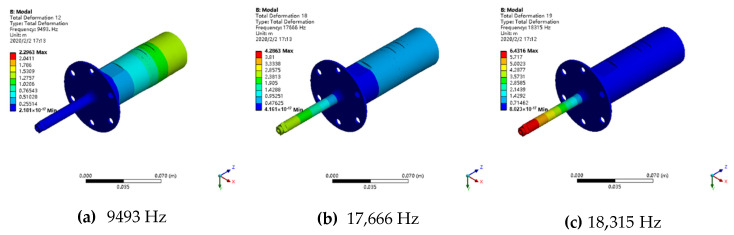
Modal shape of the first three longitudinal vibrations. (**a**) 9493 Hz; (**b**) 17,666 Hz; (**c**) 18,315 Hz.

Modal analysis is carried out to investigate the natural frequency and vibration shape of the turning system. The front face of the flange is set to be fixed to ground. After setting the contact region and meshing, 25 order resonant frequencies were calculated as shown un Figure 3. At the 12th, the 18th and the 19th modal the horn vibrates longitudinally, their resonant frequencies are 9493 Hz, 17,666 Hz and 18,315 Hz. Figure 4 describes these three modal shapes. As presented in figure, the 19th vibration mode is in positive agreement with requirements because it outputs the largest relative deformation, and its maximum vibration occurs at the front end of the horn, whereas the back end and the transducer only vibrates slightly. More detailed analysis will be examined in harmonic response analysis.

**Figure 5:**
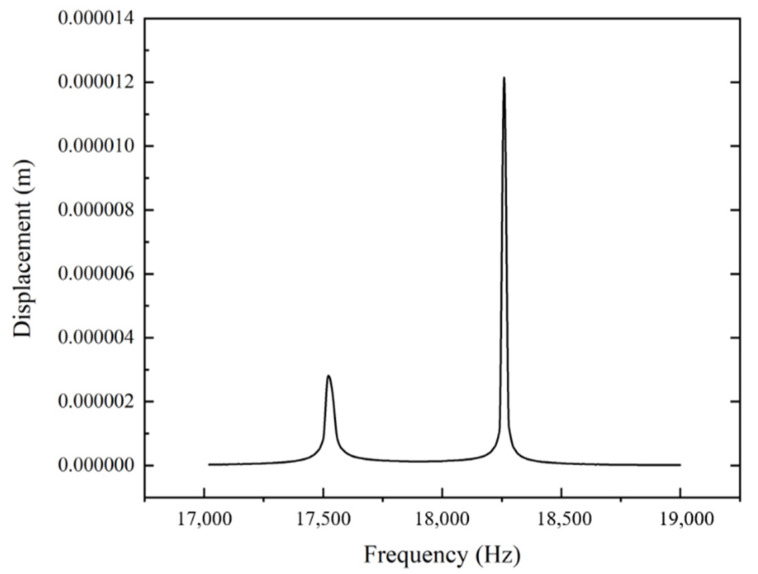
Displacement curve of the tool between 17,000 Hz and 19,000 Hz.

In the modal analysis, the vibration mode is relative value, which cannot reflect the true amplitude of the ultrasonic turning device at the tool. It is crucial to further analyze the harmonic response of the device on the basis of the 19th mode to obtain the amplitude at the tool tip. The harmonic response analysis is then conducted by workbench. Remote force is applied along the horn direction on its back end. The sweep range is set to between 17,000 Hz and 19,000 Hz (based on the 19th mode) with 100 solution intervals. Figure 5 shows the output displacement curve of the setting frequencies regarding the turning tool installed on the top of the horn. It could be observed that near to the frequency of 18,260 Hz, the structure is in the state of resonance, where the output displacement reaches the peak as 12 μm.

The designed LUAT system can output a considerable vibration with an amplitude up to 12 μm and a frequency of 18,260 Hz in the 19th mode, which is suitable for the turning process.

### 2.3. Vibration Testing Experiment of the LUAT System

**Figure 6:**
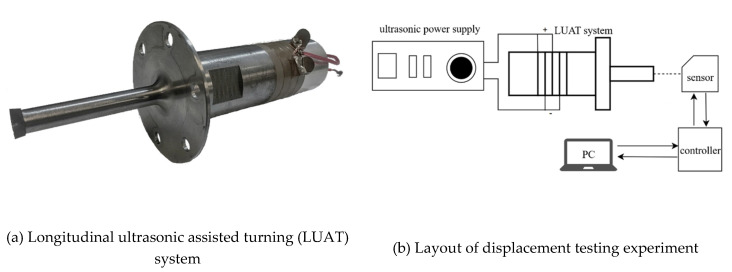

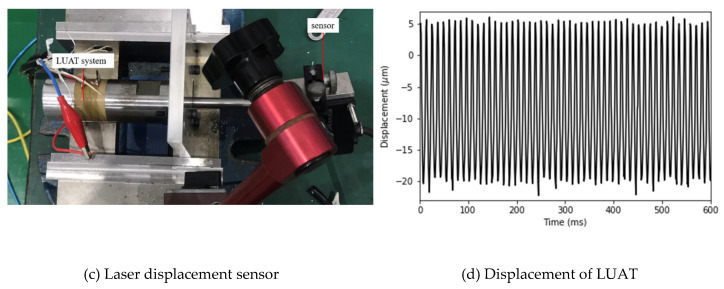
Displacement testing experiment. (**a**) longitudinal ultrasonic assisted turning (LUAT) system; (**b**) layout of displacement testing experiment; (**c**) laser displacement sensor; (**d**) displacement of LUAT.

A vibration testing experiment is conducted to verify the designed LUAT system, as shown in Figure 6. The LUAT system is activated by ultrasonic power supply BP6140. Tuning the frequency of the power supply so that the LUAT system reaches the resonant frequency, 18,265 Hz, which outputs the largest amplitude of vibration. Laser displacement sensor KEYENCE LK-H020 (KEYENCE CORPORATION, Osaka, Japan) is used to measure the displacement of the tool tip installed at the end of the ladder horn. Figure 6d depicts the displacement of the measured system, the amplitude can be calculated as around 12 μm. The result of the experiment verifies the structure design and the simulations of the LUAT system.

## 3. Turning Simulation of Vit1 Bulk Metallic Glass

### 3.1. Finite Element Modeling of Vit1 BMG Turning Simulation

The accuracy of material model has a great influence on the success of simulation. In order to accurately reflect the nonlinear problem in the cutting process, it is necessary to comprehensively consider the stress–strain state of each point in the BMG under the complex strain state. The constitutive model of the material represents the stress–strain relationship of the material under load and describes it by mathematical expression. D–P model is a constitutive model of geotechnical materials. It not only considers the effect of intermediate principal stress on material yield, but also explains the experimental phenomena of “shear expansion effect” and “tension compression asymmetry” of metallic glass [8,23]. In this study, the D–P model is used as the constitutive model of vit1-metallic glass, and its main parameters [14,24] are shown in Table 3. Specifically, the D–P plasticity is defined by dilatancy angle, friction angle and flow–stress ratio, d-p hardening is defined by yield stress and absolute plastic strain.

The material parameters of vit1 BMG and turning tool are shown in Table 4. The constitutive model of metallic glass and the physical parameters of material are introduced into ABAQUS (Dassault Systèmes, Vélizy-Villacoublay Cedex, France), and the chip separation criterion is shear criterion.

**Figure 7:**
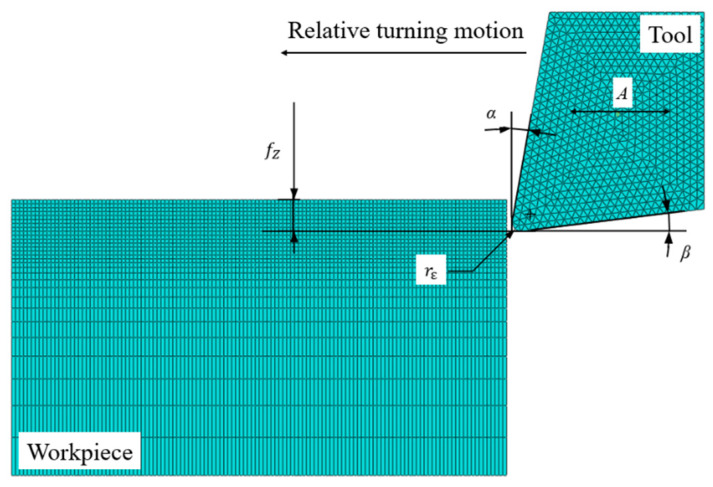
Parameters and meshing of BMG turning simulation.

Figure 7 shows the parameters and meshing of BMG turning simulation. YG-8 cemented carbide tool with 10° rake angle *α* and 7° relief angle *β* is chosen, and the nose radius $r_{\varepsilon }$ is set as 0.2 mm. The longitudinal ultrasonic vibration is applied to the tool in the amplitude *A* paralleling to the relative turning motion. Due to the short cutting distance, the deformation of the turning tool could be ignored, and the tool is then set as a rigid body. In order to reduce the amount of calculation and ensure the accuracy of calculation, the side grid close to the cutting surface is relatively dense, and the side grid of the principle cutting surface is relatively sparse.

### 3.2. Analysis of LUAT Process

In conventional turning (CT), the absolute speed of the tool tip in the peripheral direction of the workpiece is a fixed value. The introduction of ultrasonic vibration triggers the tool tip to produce high frequency reciprocating vibration in the peripheral direction. The average speed in a cycle $\bar{v_{u}}$ can be obtained from Equation (4):

(4) $$\bar{v_{u}}=2\times A\times f$$

where, *A* and *f* represent the amplitude and the frequency of the tool tip vibration.

According to the modal analysis and harmonic response analysis, the suitable resonant frequency *f* is 18,260 Hz, and the displacement *A* is 12 μm. Consequently, the average speed $\bar{v_{u}}$ = 438.24 mm/s. The turning motion is equivalent to the fixed workpiece and the moving tool. At this time, the resultant velocity v can be regarded as the superposition of the workpiece velocity $v_{w}$ and the instantaneous velocity of the tool $v_{u}$ which comes from ultrasonic vibration.

(5) $$v=v_{w}+v_{u}$$

Let $v_{u}$ be cosine function and $v_{u}=a\cos\omega t$, then there is:

(6) $$\bar{v_{u}}\times t=\int_{0}^{t}a\cos\omega tdt$$

Take t as half of the ultrasonic vibration period, then:

(7) $$a=\frac{\bar{v_{u}}\times t\times \omega }{1-\cos\omega t}$$

Plugging ω=2πf, it can be easily obtained that a=688.04 mm/s. Therefor $v_{u}=688.04\cos114,731t$, at this time the critical turning speed $v_{t}=\underset{}{\max}v_{u}=688.04$ mm/s.

The relationship between tool tip and workpiece is affected by the critical turning speed $v_{u}$ of tool tip and workpiece velocity $v_{w}$. Specifically,

- When $v_{w}$ is much larger than $v_{u}$, the direction of the closing speed v remains unchanged, and the size is first equal to $v_{w}$, which can be regarded as CT;
- When $v_{w}$ is slightly larger than $v_{u}$, the tool tip keeps contact with the workpiece, the direction of closing speed remains unchanged and the size changes periodically;
- When $v_{w}$ is smaller than the maximum value of $v_{u}$, the direction of closing speed changes periodically, and the contact separation state of tool tip and workpiece presents high frequency. At this time, the relationship between the tool and the workpiece is changed from the original tool extruding the workpiece to the high frequency tool hitting the workpiece.

In the range of 100 mm/s to 1800 mm/s, different $v_{w}$ are selected to carry out the turning simulation experiment of BMG. The interval is 100 mm/s when $v_{w}$ is below 600 mm/s and then rise to 200 mm/s when $v_{w}$ is above 600 mm/s.

## 4. Results and Discussion

The turning simulation was conducted in different turning speeds by LUAT and CT, respectively in ABAQUS. Then the average cutting force, the instantaneous cutting force and the maximum von Mises stress are compared and analyzed.

### 4.1. Analysis of Average Cutting Force

**Figure 8:**
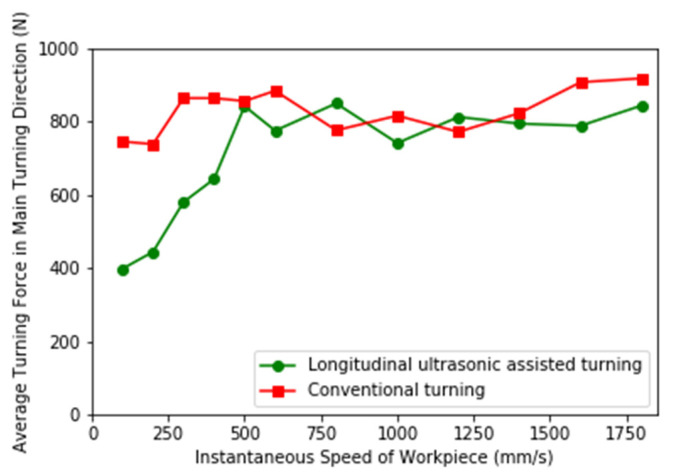
Average cutting force of LUAT and conventional turning (CT).

The cutting force of the main turning direction is extracted from 50 sampling points when the tool is turned for 5 mm for analysis. The average cutting force of the LUAT and the CT at different $v_{w}$ is shown in Figure 8.

It can be observed from figure that when $v_{w}$ is small, the average cutting force of LUAT is significantly smaller than that of CT, and the smaller $v_{w}$ is, the more obvious the reduction of the average cutting force of longitudinal vibration is. When $v_{w}$ is close to $v_{t}$, the effect of longitudinal ultrasonic vibration is gradually reduced. When $v_{w}$ is greater than $v_{t}$, the turning force of LUAT has no obvious reduction compared with CT.

**Figure 9:**
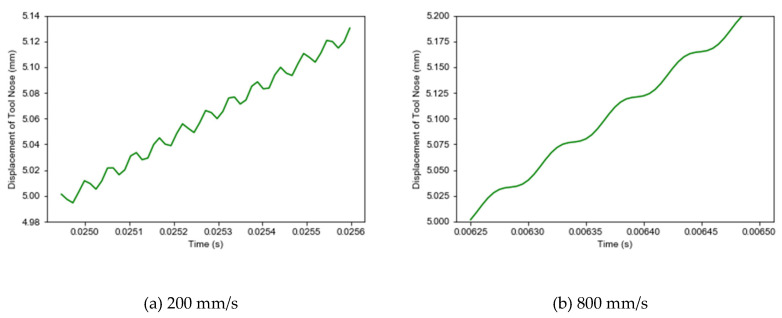
Displacement trajectory of the tool tip. (**a**) 200 mm/s; (**b**) 800 mm/s.

The reason for this phenomenon can be the contact-separation phenomenon between the tool tip and the workpiece. In the process of LUAT, the displacement where the tool nose cuts into the workpiece is extracted, and the displacement curve when the speed is 200 mm/s and 800 mm/s is shown in Figure 9. Where (a) is the displacement curve at 200 mm/s, when $v_{w}$ is less than $v_{t}$, the direction of resultant velocity changes periodically under longitudinal vibration, and the tip of the tool contacts the workpiece discontinuously.

When $v_{w}$ < $v_{t}$, the duty cycle $\alpha =t_{1}/T$, where $t_{1}$ represents the time when the tool movement has the same direction with the turning direction in a cycle and T is the cycle. From Equation (5), we can get,

(8) $$t_{1}=\frac{1}{\pi f}\cos^{-1}\left(-\frac{v_{w}}{v_{t}}\right)$$

Then,

(9) $$\alpha =\frac{1}{\pi }\cos^{-1}\left(-\frac{v_{w}}{v_{t}}\right)$$

It can be seen from Equation (9) that α decreases with the decrease of $v_{w}/v_{t}$, and the decrease of instantaneous speed $v_{w}$ of workpiece will increase the time when the resultant velocity is opposite to the main cutting speed in a cycle, and the time ratio of tool tip backward increases in displacement. The cutting force decreases significantly when the tool tip recedes, and then the average cutting force decreases.

### 4.2. Analysis of Instantaneous Cutting Force

**Figure 10:**
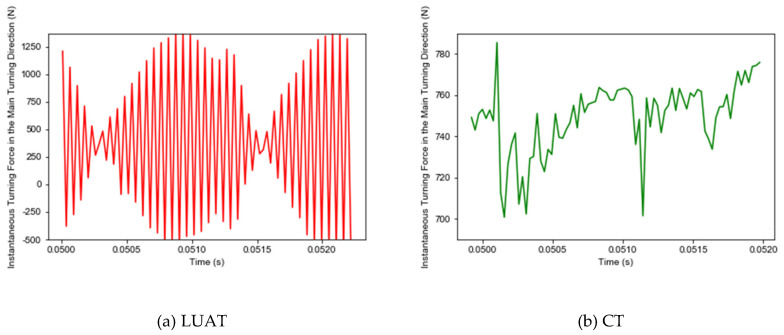
Instantaneous cutting force in main turning direction. (**a**) LUAT; (**b**) CT.

In LUAT and CT, 80 sampling points are, respectively extracted when the tool is turning 5 mm under the condition of $v_{w}$ = 100 mm/s. The instantaneous cutting force in the main turning direction is compared in Figure 10.

**Figure 11:**
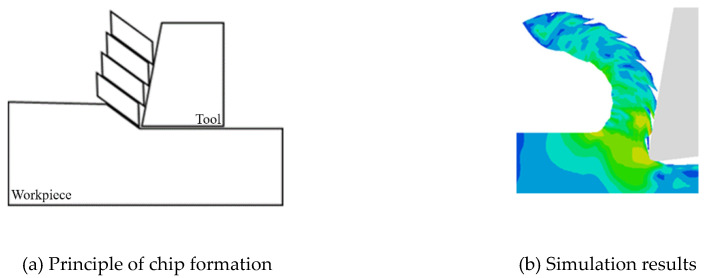
Formation of BMG chips. (**a**) Principle of chip formation; (**b**) simulation results.

During the CT process, the tool extrudes the workpiece to deform, the stress and strain continue to accumulate until the workpiece material fails—this is how the chips generate. Conversely, as shown in Figure 11, due to the periodic contact separation between tool and workpiece, the stress form of workpiece changes from extrusion to high-frequency impact, and the peak value of instantaneous cutting force is significantly larger than that of CT.

Furthermore, due to the contact-separation phenomenon, the periodic change of cutting force with chip breaking is more obvious. In CT, when the tool contacts the workpiece continuously, the BMG is still squeezed by the tool when the chip generates. In contrast, during the LUAT, the larger instantaneous cutting force and the impact of the tool under the contact separation state are more conducive to the failure of BMG, which promotes the generation of chips.

### 4.3. Internal Stress Analysis of Workpiece

**Figure 12:**
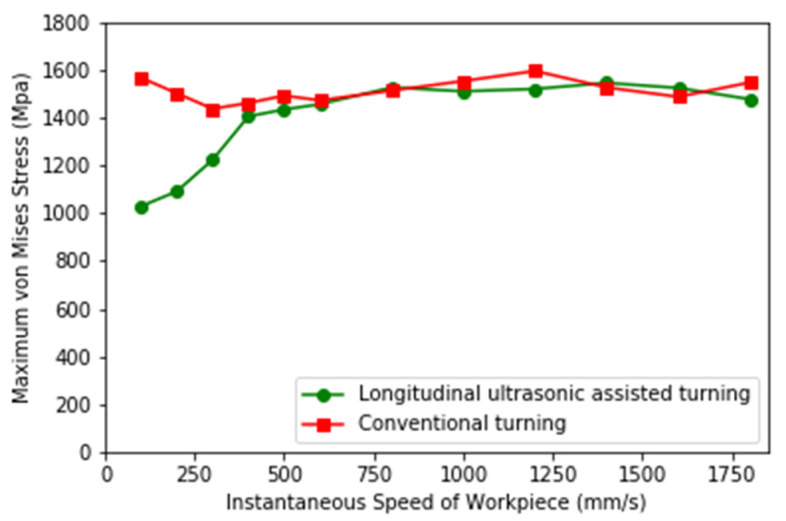
Maximum Mises stress of LUAT and CT.

The von Mises stress field obtained at different workpiece speeds from the simulation of LUAT and CT is shown in Figure 12. It can be noticed that in low-speed turning, the maximum von Mises stress of LUAT is significantly smaller than that of CT. With the increase of workpiece speed, the machining stress of LUAT increases gradually. In high-speed turning, the machining stress of LUAT is similar to that of CT. This is consistent with the observation from the average turning force above. Lower average turning force has an effect on von Mises stress in low speed LUAT. In addition, taking the contact-separate phenomenon into account, the relationship between the tool and the workpiece changes from continuously extrusion to discontinuously impact. The lower the turning speed is, the longer time the tool is separate to the workpiece in a single period, thus the materials in between can spring-back and release the stress. However, when the turning speed goes up, as the separated time decrease, the turning process becomes increasing continuously, hence the spring-back no longer happen. In short, the effect of longitudinal ultrasonic vibration only works at low turning speed when turning the BMG.

**Figure 13:**
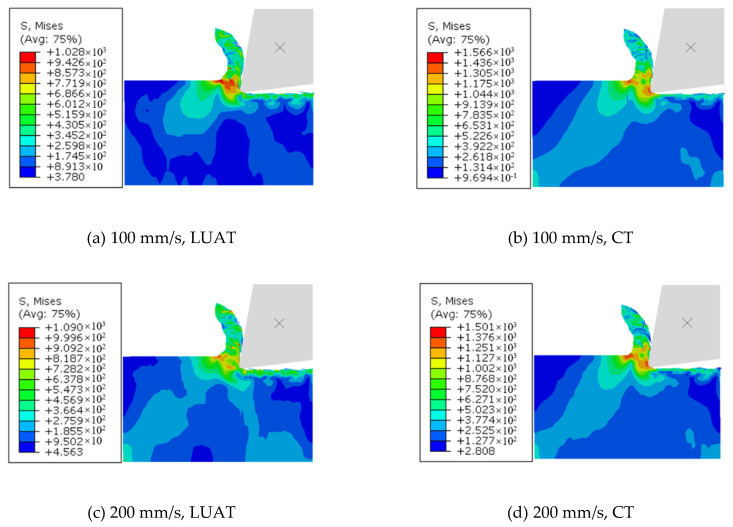

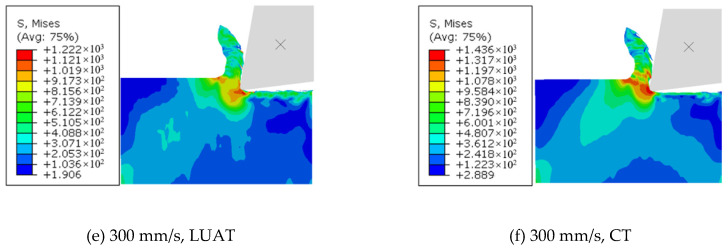
Maximum von Mises stress of LUAT and CT. (**a**) 100 mm/s, LUAT (**b**) 100 mm/s, CT; (**c**) 200 mm/s, LUAT (**d**) 200 mm/s, CT; (**e**) 300 mm/s, LUAT (**f**) 300 mm/s, CT.

Figure 13 depicts the von Mises stress nephogram of LUAT and CT at different turning speed in BMG turning. As depicted in figure, the maximum stress by LUAT in the first deformation area is obviously smaller than that by CT. Due to the phenomenon of discontinuous contact, the local deformation of the contact surface changes from extrusion to high-frequency impact, accordingly the stress can be released during separation. This results in the decrease of shear slip deformation and work hardening in the first deformation zone of the BMG. It can be easily observed that there is less residual stress in machined surface in LUAT, which represents less extrusion and friction during the turning process, given that the crystallization or even phase transition of BMG can be mitigated.

**Figure 14:**
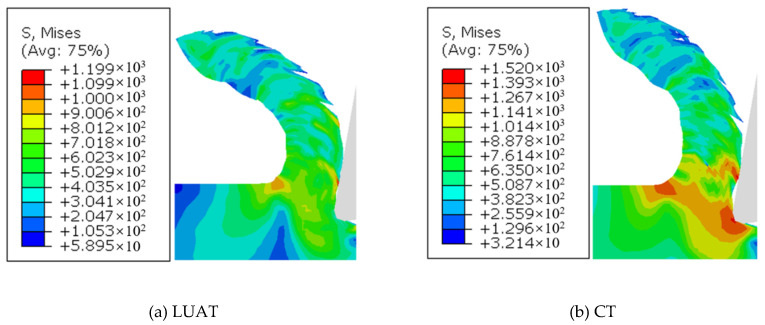
Shapes and von Mises stress in chips. (**a**) LUAT; (**b**) CT.

Shapes and Von Mises stress in chips of LUAT and CT are depicted in Figure 14, respectively. During turning process, the BMG would be softened in the crack areas in chips, which can damage the tool and reduce the machined surface quality. In the case of LUAT, the residual stress in the chip is less than that in CT, which can improve the chip integrity. In addition, due to the promoting effect of chip formation, the chip of LUAT is more curved than that of CT, which is beneficial for releasing the residual stress, promoting chip removal during the turning process and improving the quality of machining.

## 5. Conclusions

To improve the turning performance of BMG, first, a longitudinal ultrasonic vibration turning system was designed and then relative modal and harmonic response analysis was conducted. The analyses indicated that the vibration shape of the system met the design requirements at 19th mode. Through the experimental test, it turned out that the vibration amplitude of the system can reach 12 μm, which verified the design and the simulation. Therefore, the established LUAT system can realize ultrasonic assisted turning. Then the critical turning speed was derived, which proved a key factor of the effect of ultrasonic vibration and is determined by the parameters of the turning system. Finally turning simulations in different turning speeds were conducted on Vit1 BMG described by D–P constitutive model. For this work, the following conclusions can be drawn:

1. Simulation and theoretical analysis show that there is a critical turning speed in LUAT, which is determined by the longitudinal ultrasonic device. When the turning speed is lower than the critical turning speed, the longitudinal ultrasonic vibration can effectively reduce the average cutting force and von Mises stress of BMG cutting processing;
2. When the longitudinal ultrasonic vibration is applied in low speed turning, the tool and workpiece will have contact-separation phenomenon, which is conducive to the reduction of average cutting force and the periodic formation of chips;
3. During the turning process of BMG, the application of longitudinal ultrasonic vibration is rewarding to the formation of chips, so that the periodicity of instantaneous turning force is more obvious, which can help to form more regular chips and machined surface morphology;
4. LUAT can remarkably decrease the von Mises stress in both chips and machined surface, especially in the first deformation zone. This reduction is beneficial to improve the turning process of BMG.

## Figures and Tables

**Table 1:** Parameters of piezoelectric ceramics.

Material	Poisson’s Ratio (−)	Density (kg/m^3^)	Young’s Modulus (GPa)
PZT-8	0.31	7600	77

**Table 2:** Parameters of 1045 steel.

Material	Longitudinal Wave Velocity (mm/s)	Density (kg/m^3^)	Elastic Modulus (GPa)
1045 steel	5.17 × 10^6^	7890	21

**Table 3:** Related parameters of linear D–P model of BMG.

Material	Dilatancy Angle (°)	Friction Angle (°)	Flow–Stress Ratio (−)	Yield Stress (MPa)	Abs Plastic Strain (−)
Vit1	11.902	11.902	1	1194.62	0

**Table 4:** Physical parameters of Bulk metallic glass (BMG) and turning tool.

Material	Poisson’s Ratio (−)	Density (kg/m^3^)	Young Modulus (GPa)
Vit1	0.36	6125	96,000
YG-8	0.22	14,700	640,000

## References

[1] Wang W.H. (2012). The elastic properties, elastic models and elastic perspectives of metallic glasses. Prog. Mater. Sci..

[2] Zhu P.-Z., Qiu C., Fang F.-Z., Yuan D.-D., Shen X.-C. (2014). Molecular dynamics simulations of nanometric cutting mechanisms of amorphous alloy. Appl. Surf. Sci..

[3] Hofmann D.C. (2013). Bulk metallic glasses and their composites: A brief history of diverging fields. J. Mater..

[4] Chen M.W. (2011). A brief overview of bulk metallic glasses. NPG Asia Mater..

[5] Liens A., Etiemble A., Rivory P., Balvay S., Pelletier J.M., Cardinal S., Fabregue D., Kato H., Steyer P., Munhoz T. (2018). On the Potential of Bulk Metallic Glasses for Dental Implantology: Case Study on Ti40Zr10Cu36Pd14. Materials.

[6] Basak A.K., Zhang L.C. (2018). Deformation of Ti-Based Bulk Metallic Glass Under a Cutting Tip. Tribol. Lett..

[7] Browne D.J., Stratton D., Gilchrist M.D., Byrne C.J. (2013). Bulk Metallic Glass Multiscale Tooling for Molding of Polymers with Micro to Nano Features: A Review. Metall. Mater. Trans. A Phys. Metall. Mater. Sci..

[8] Zhao Y., Lu J., Zhang Y., Wu F., Huo D. (2016). Development of an analytical model based on Mohr–Coulomb criterion for cutting of metallic glasses. Int. J. Mech. Sci..

[9] Karaguzel U., Bakkal M. Orthogonal cutting of Zr based bulk metallic glass by radial turning. Proceedings of the International Conference On Materials Science, Metal and Manufacturing.

[10] Fujita K., Morishita Y., Nishiyama N., Kimura H., Inoue A. (2005). Cutting Characteristics of Bulk Metallic Glass. Mater. Trans..

[11] Bakkal M., Liu C.T., Watkins T.R., Scattergood R.O., Shih A.J. (2004). Oxidation and crystallization of Zr-based bulk metallic glass due to machining. Intermetallics.

[12] Bakkal M., Shih A.J., McSpadden S.B., Liu C.T., Scattergood R.O. (2005). Light emission, chip morphology, and burr formation in drilling the bulk metallic glass. Int. J. Mach. Tools Manuf..

[13] Bakkal M., Shih A.J., Scattergood R.O., Liu C.T. (2004). Machining of a Zr–Ti–Al–Cu–Ni metallic glass. Scr. Mater..

[14] Bakkal M., Shih A.J., Scattergood R.O. (2004). Chip formation, cutting forces, and tool wear in turning of Zr-based bulk metallic glass. Int. J. Mach. Tools Manuf..

[15] Liu C.T., Heatherly L., Easton D.S., Carmichael C.A., Schneibel J.H., Chen C.H., Wright J.L., Yoo M.H., Horton J.A., Inoue A. (1998). Test environments and mechanical properties of Zr-base bulk amorphous alloys. Metall. Mater. Trans. A Phys. Metall. Mater. Sci..

[16] Lauwers B., Bleicher F., Ten Haaf P., Vanparys M., Bernreiter J., Jacobs T., Loenders J., Leuven K. Investigation of the Process-Material Interaction in Ultrasonic Assisted Grinding of ZrO2 based Ceramic Materials. Proceedings of the 4th CIRP International Conference on High Performance Cutting.

[17] Pujana J., Rivero A., Celaya A., de Lacalle L.N.L. (2009). Analysis of ultrasonic-assisted drilling of Ti6Al4V. Int. J. Mach. Tools Manuf..

[18] Sui H., Zhang X.Y., Zhang D.Y., Jiang X.G., Wu R.B.A. (2017). Feasibility study of high-speed ultrasonic vibration cutting titanium alloy. J. Mater. Process. Technol..

[19] Maurotto A., Muhammad R., Roy A., Silberschmidt V. (2013). Enhanced ultrasonically assisted turning of a β-titanium alloy. Ultrasonics.

[20] Nath C., Rahman M., Neo K.S. (2009). A study on the effect of tool nose radius in ultrasonic elliptical vibration cutting of tungsten carbide. J. Mater. Process. Technol..

[21] Luo F., Sun F., Li K.S., Gong F., Liang X., Wu X.Y., Ma J. (2018). Ultrasonic assisted micro-shear punching of amorphous alloy. Mater. Res. Lett..

[22] Ma J., Liang X., Wu X., Liu Z., Gong F. (2015). Sub-second thermoplastic forming of bulk metallic glasses by ultrasonic beating. Sci. Rep..

[23] Schuh C.A., Lund A.C. (2003). Atomistic basis for the plastic yield criterion of metallic glass. Nat. Mater..

[24] Lu J., Ravichandran G., Johnson W.L. (2003). Deformation behavior of the Zr41.2Ti13.8CU12.5Ni10Be22.5 bulk metallic glass over a wide range of strain-rates and temperatures. Acta Mater..

